# Can we conclude a potential therapeutic action for Parkinson’s disease by using postmortem tissue and a preclinical model based on an exogenous neurotoxin?

**DOI:** 10.1038/s41419-018-0798-0

**Published:** 2018-07-03

**Authors:** Juan Segura-Aguilar

**Affiliations:** Molecular & Clinical Pharmacology ICBM Faculty of Medicine, University of Chile, Santiago, Chile

One of the major problems in the translation of basic research into clinical studies and new therapies is the preclinical model used to conclude a possible therapeutic action. Recently, an excellent paper has been published in *Cell Death and Disease* by Leem et al. 2018,^1^ who found that “the sustained level of astrocyte elevated gene-1 (AEG-1) as an important anti-apoptotic factor in nigral DA neurons might potentiate the therapeutic effects of treatments, such as Rheb(S16H) administration, on the degeneration of the dopamine (DA) pathway that characterizes PD”^1^. In my opinion, these important results require more discussion about the potential use of the aforementioned approach in Parkinson’s disease treatment. The authors’ conclusion is based on postmortem material, and 6-hydroxydopamine as a preclinical model.

The postmortem substantia nigra of patients with Parkinson’s disease includes neurons and glia cells, which survive the degenerative process over time. We have to remember that motor symptoms appear after many years of neurodegeneration^2^ and the progression of the disease is also very slow in the long run. The neurons or glia cells that die during the degenerative process are removed by macrophage, with the postmortem material containing only neurons and glia cells that resisted this process. Therefore, it is not clear whether the remaining neurons that survive the generative process represent the neurons lost during the degenerative process over time. Another controversial interpretation of postmortem studies has been the role of Lewy bodies in Parkinson’s disease. Lewy bodies are found, in the case of Parkinson’s disease, in the postmortem brain, with alpha-synuclein fibrils among the major components of these abnormal proteins’ aggregation^3^. Alpha-synuclein aggregation into neurotoxic oligomers seems to be involved in the neurodegenerative process in Parkinson’s disease, but alpha-synuclein aggregation into fibrils is a non-toxic pathway. Therefore, the formation of Lewy bodies seems to be a protective mechanism in Parkinson’s disease^4^.

Studies have been undertaken with 6-hydroxydopamine animals as preclinical models for Parkinson’s disease, where AEG-1 expression was increased by using an adeno-associated virus as vector^1^. A decrease in the 6-OHDA-dependent apoptotic death of dopaminergic neurons was accompanied with an increase in the expression of AEG-1^1^. However, the validity of preclinical models for Parkinson’s disease in the case of 6-hydroxydopamine and other exogenous neurotoxins (1-methyl-4-phenyl-1,2,3,6-tetrahydropyridine (MPTP) and rotenone) has been questioned, due to the significant volume of failed clinical studies based on exogenous neurotoxins^5^. The reason for this could be the fact that exogenous neurotoxins do not replicate what happens with the disease. The degeneration of nigrostriatal dopaminergic neurons containing neuromelanin is a very slow process, such that many years pass before motor symptoms appear^2^; the progression of the disease is also very slow (Braak). In contrast, exogenous neurotoxins induce very rapid and massive cell loss in animals^6^. Meanwhile, in humans, the exogenous neurotoxin MPTP induced severe Parkinsonism after just three days in drug addicts who consume a synthetic drug contaminated with MPTP^7^.

These results suggest that an exogenous neurotoxin cannot be involved in the degeneration of the nigrostriatal system in Parkinson’s disease, and therefore the use of exogenous neurotoxins as preclinical models seems to be a waste of time. In general, the scientific community agrees that the loss of dopaminergic neurons from the nigrostriatal system involves mitochondrial dysfunction, the aggregation of alpha-synuclein into neurotoxic oligomers, protein degradation dysfunction of both lysosomal and proteasomal systems, neuroinflammation, and oxidative and endoplasmic reticulum stres^8–10^. However, the question concerns what triggers these mechanisms. The overwhelming slowness of the degenerative process, which takes many years in Parkinson’s disease, suggests that an endotoxin generated within dopaminergic neurons containing neuromelanin could trigger all these mechanisms.

Possible endogenous neurotoxins are alpha-synuclein oligomers, which induce mitochondrial dysfunction, inhibit both autophagy and proteasomal activity, induce neuroinflammation, increase oxidative stress and induce disturbance in the endoplasmic reticulum and Golgi traffic^11^. In addition, it has been proposed that alpha-synuclein neurotoxic oligomers can be released from the neuron, thus disseminating its neurotoxic action to other neurons or glia cells^12^. However, the problem with this hypothesis concerns (i) what induces alpha-synuclein aggregation into neurotoxic oligomers inside of the dopaminergic neurons containing neuromelanin; and (ii) the case where the propagation of alpha-synuclein is involved in the degeneration of the nigrostriatal system, which means that the development and progression of the disease should be a very rapid process, contrasting with the extremely slow progression of Parkinson’s disease. It has been proposed that the endogenous neurotoxin can be involved in the degeneration of the nigrostriatal neurons in Parkinson’s disease^6^.

**Fig. 1:**
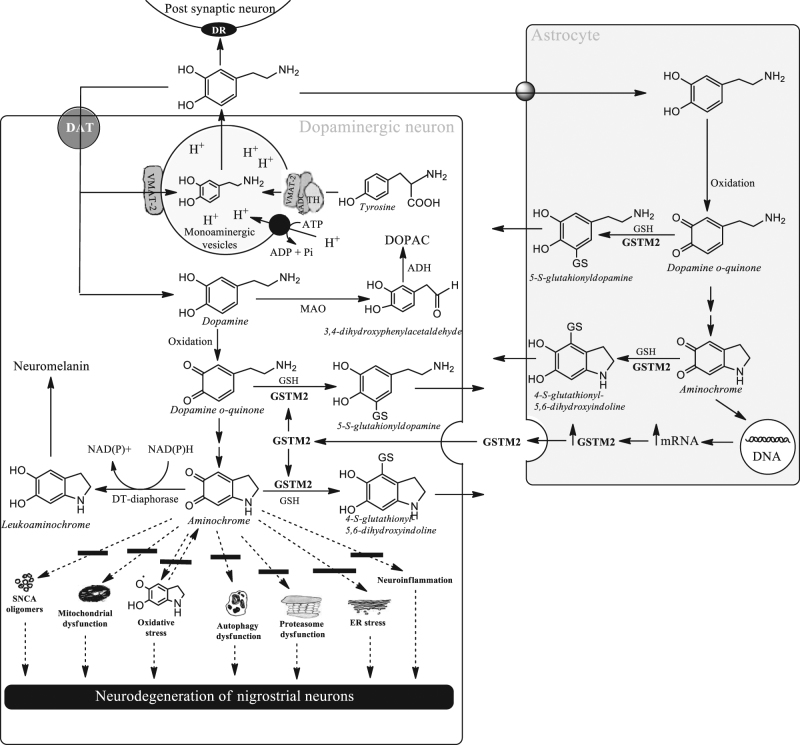
Aminochrome neurotoxicity and protective reactions. In dopaminergic neurons, tyrosine is converted into L-dopa by tyrosine hydroxylase, forming a kind of complex with amino acid decarboxylase (AADC) and vesicular monoamine transporter-2 (VMAT-2), which is attached to the membrane of postsynaptic monoaminergic vesicles. L-dopa, inside of this complex, is converted into dopamine by AADC, which is immediately transported to the vesicles by VMAT-2. The low pH found inside the vesicles stabilizes the dopamine, such that it becomes completely stable and can be stored at high levels of concentration, preventing dopamine oxidation into ortho-quinones at a physiological pH in the cytosol. Released dopamine from dopaminergic neurons binds the dopamine receptors in the postsynaptic neurons. Later, free dopamine in the inter-synaptic space experiences the following: (i) The reuptake of dopamine is performed by the dopamine transporter (DAT), resulting in dopaminergic neurons where dopamine can be transported to the monoaminergic vesicles by VMAT-2 or can be oxidized in the cytosol. Dopamine can be degraded to 3,4-dihydroxyphenylacetaldehyde by monoamine oxidase (MAO), using a molecule of oxygen and water with a concomitant formation of NH_3_ and H_2_O_2_. Alternatively, free dopamine in the cytosol oxidizes into dopamine *o*-quinone, which immediately cyclizes into aminochrome. Aminochrome can be neurotoxic by inducing the formation of neurotoxic oligomers, inducing mitochondrial dysfunction, oxidative stress, autophagy dysfunction, proteasome dysfunction, endoplasmic reticulum stress, and neuroinflammation. However, aminochrome neurotoxicity can be prevented by the two-electron reduction of aminochrome catalyzed by DT-diaphorase into leukoaminochrome, which finally forms neuromelanin in the dopaminergic neurons. (ii) Astrocytes take up the remaining dopamine, which oxidizes into dopamine *o*-quinone, while GSTM2 conjugates the dopamine *o*-quinone into 5-glutathionyl dopamine, which can later be converted into 5-cysteinyl dopamine, a stable product. Aminochrome can also be conjugated by GSTM2 into 4-S-glutathionyl-5,6-dihydroxyindoline, which is resistant to biological oxidizing agents, such as dioxygen, superoxide and hydrogen peroxide. Interestingly, aminochrome induces the overexpression of GSTM2, which can be secreted from astrocytes into the inter-synaptic space where dopaminergic neurons can internalize into the neuron cytosol. GSTM2, in the dopaminergic neuron cytosol, conjugates with glutathione both dopamine *o*-quinone and aminochrome. Both DT-diaphorase and GSTM2 protect the dopaminergic neurons by preventing aminochrome-induced neurotoxicity

Aminochrome induces the formation of alpha-synuclein neurotoxic oligomers, mitochondria dysfunction, neuroinflammation, protein degradation dysfunction of both lysosomal and proteasomal systems, and oxidative and endoplasmic reticulum stress^8–10, 13^ (Fig. 1). Aminochrome is a metabolite formed during dopamine oxidation into neuromelanin, which is a harmless pathway because healthy senior brains keep their dopaminergic neurons containing neuromelanin intact^14^. There is something paradoxical about the fact that aminochrome neurotoxin is formed along a completely normal and harmless pathway. Dopamine oxidation into neuromelanin is a harmless pathway due to the existence of two enzymes, which prevent aminochrome neurotoxicity: (i) DT-diaphorase, which catalyzes the two-electron reduction of aminochrome and is expressed in dopaminergic neurons and astrocytes^9^; and (ii) glutathione transferase M2-2 (GSTM2), which catalyzes the glutathione conjugation of both aminochrome and its precursor dopamine *o*-quinone. Aminochrome conjugate is resistant to biological oxidizing agents, such as dioxygen, superoxide, and hydrogen peroxide. The conjugate of dopamine *o*-quinone (5-glutathionyl dopamine) is normally degraded to 5-cysteinyl dopamine, which has been detected in human cerebrospinal fluid and neuromelanin^9^. GSTM2 is only expressed in astrocytes, but this enzyme protects against aminochrome toxicity, both in astrocytes and in dopaminergic neurons. Astrocytes secrete GSTM2, with dopaminergic neurons internalizing this enzyme into the cytosol protecting the neuron against aminochrome-induced cell death^11^.

Aminochrome has been proposed as a new preclinical model, which, unlike neurotoxins, does not induce rapid and massive cell death, but rather neuronal dysfunction. A single dose of aminochrome induces: (i) contralateral rotation without a significant loss of tyrosine hydroxylase positive neurons; (ii) a significant decrease in dopamine release due to a significant decrease in the number of monoaminergic vesicles; (iii) a significant decrease in presynaptic vesicles as a consequence of mitochondrial dysfunction that lowers ATP levels; (iv) a significant decrease in dopamine levels with a significant increase in GABA levels; and (v) cell shrinkage, suggesting neuronal dysfunction^15^.

In conclusion, the interpretation of results obtained with the postmortem substantia nigra of patients with Parkinson’s disease and preclinical studies with 6-hydroxydopamine is controversial; therefore, further discussion is needed on this issue.
